# Vascular health and exercise in females throughout the lifespan: Exploring puberty, pregnancy and menopause

**DOI:** 10.1113/EP092170

**Published:** 2025-01-30

**Authors:** Kathleen B. Miller, M. Erin Moir, Brandon G. Fico

**Affiliations:** ^1^ Department of Health and Exercise Science, Morrison Family College of Health University of St. Thomas Saint Paul Minnesota USA; ^2^ Bruno Balke Biodynamics Laboratory, Department of Kinesiology University of Wisconsin‐Madison Madison Wisconsin USA; ^3^ Department of Exercise Science and Health Promotion Florida Atlantic University Boca Raton Florida USA

**Keywords:** aerobic, arterial stiffness, endothelial function, oestrogen, progesterone, women

## Abstract

This narrative review highlights the impact of exercise on vascular health in females over the lifespan with an emphasis on puberty, pregnancy and menopause. These events encompass substantial changes in sex hormone levels, particularly oestrogens and progesterone. They are also accompanied by distinct adaptations of the central, peripheral and cerebral vasculature. Regular exercise is an effective mechanism to reduce vascular risk in females of all ages, especially for those at higher risk for vascular disorders. However, there are large variabilities in the vascular adaptations to exercise in females that may be related to circulating sex hormone levels. In addition, exogenous hormones, such as oral contraceptives taken after puberty or hormonal replacement therapy taken to mitigate symptoms of menopause, may interact with exercise‐induced changes in vascular function. We highlight how more research is needed to understand the optimal exercise interventions to promote vascular health in females across the lifespan, especially during times of hormonal transition.

## Abstract (highlights)

- **What is the topic of this review?**
  This narrative review highlights the impact of exercise on vascular health in females over the lifespan with an emphasis on puberty, pregnancy and menopause.
- **What advances does it highlight?**
  Throughout the lifespan, females may experience substantial fluctuations in sex hormones related to puberty, pregnancy or menopause that influence vascular structure and function. Habitual exercise is associated with reduction of cardiovascular disease risk in females of all ages. However, there is a large amount of variability in vascular adaptations to habitual exercise, which may be dependent on sex hormone fluctuations.

## 1 INTRODUCTION

Cardiovascular diseases (CVD) are the number one cause of mortality and morbidity in developing nations (Lakatta, 2002). Regular exercise has been demonstrated to reduce vascular risk in all adults by improving vascular function or slowing age‐related changes in vascular reserve. Yet, the impact of regular exercise on vascular health in females, who undergo large fluctuations in the sex hormones oestrogens and progesterone throughout their lifespan, is less understood. Vascular adaptations to exercise in females are highly variable and may be related to age‐, menstrual cycle‐ or pregnancy‐associated changes in hormone levels (Ji et al., 2024; Moreau et al., 2024; Stanhewicz et al., 2018). Therefore, the objective of this review is to describe the hormonal changes and the structural and functional vascular adaptations during three major phases of hormonal transition in females: puberty, pregnancy and menopause. Our second aim is to describe the impact of exercise on vascular health during these three hormonal events. Exogenous hormone use, such as oral contraceptives taken after puberty and hormonal replacement therapy taken during the menopausal transition, is also discussed. This review will focus on vascular health in the central, peripheral and cerebral circulations in healthy women without overt CVD and women with elevated CVD risk factors such as a sedentary lifestyle. We will pay particular attention to the menopausal transition, as this is a critical period for early intervention strategies to mitigate CVD risk. Our goal is to summarize the current state of knowledge regarding changes in blood pressure, arterial stiffness, endothelial function and cerebrovascular function and identify areas that warrant further investigation. Exercise adaptations in women with overt CVD, especially in the context of cardiac rehabilitation, have been reviewed elsewhere (Baig et al., 2021; Bennett et al., 2017; Szmigielska & Jegier, 2022; Witvrouwen et al., 2021).

In the context of this narrative review, the female sex is considered a biological construct defined by the structural and functional characteristics determined by sex chromosomes, specifically XX. It is acknowledged these studies do not represent the full spectrum and diversity of genders that also may undergo large endogenous or exogenous hormone changes throughout the lifespan. More research is needed that evaluates the physiology of all genders (Usselman et al., 2024).

## 2 HORMONAL CHANGES AND VASCULAR HEALTH DURING PUBERTY

### 2.1 Hormonal changes during puberty

**FIGURE 1:**
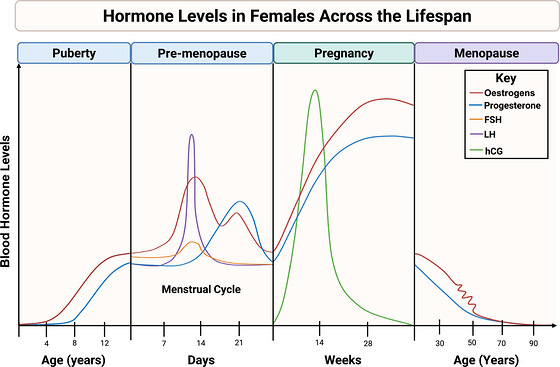
Hormonal levels in females across the lifespan. The figure shows approximate relative changes in blood hormone levels in major hormonal events of the female lifespan including puberty, the menstrual cycle, pregnancy and menopause. *x‐* and *y*‐axes are not scaled. Puberty: below age 10, total oestrogen (E1 and E2) mean levels are approximately 23 pg/mL and during puberty will increase up to 170 pg/mL. Prior to age 10, progesterone levels are approximately <10 ng/dL and then can increase to over 1000 ng/dL at the completion of puberty. Pre‐menopause: the hormone profile shown is a menstrual cycle representative of a female with a gynaecological age of greater than 2 years. Of note, at menarche onset, menstrual cycles can appear irregular, and females may experience anovulation within the first few years. Total oestrogen also fluctuates during the menstrual cycle, ranging from 60 to 200 pg/mL during the follicular phase (day 0 to 14) and 160 to 400 pg/mL during the luteal phase (day 14 to 28) with the highest levels occurring during the late follicular phase. Progesterone is lower during the follicular phase (ranging from <10 to 1563 ng/dL) and peaks during the luteal phase (ranging from 350 to 3750 ng/dL). LH ranges from 2 to 9 mIU/mL during the early follicular and late luteal phases and peaks before ovulation at approximately day 14 ranging from 18 to 49 mIU/mL. FSH ranges from 2 to 11 mIU/mL during the follicular and luteal phases and peaks mid‐cycle ranging from 6 to 35 mIU/mL. Pregnancy: during pregnancy, hCG is detectable as early as 3 weeks’ gestation ranging from 6 to 71 mIU/mL and peaks at approximately week 10–12 ranging from 27,832 to 210,612 mIU/mL, then declines throughout the remainder of pregnancy. Mean oestradiol levels range from 1.2 to 3.6 ng/mL during the first trimester (week 0–13), 5.3 to 15.1 ng/mL during the second trimester (week 14–27), and 12.8 to 32.9 ng/mL during the third trimester (week 48–40). Progesterone levels range from 17 to 40 ng/mL, 32 to 79 ng/mL and 73 to 200 ng/mL during the first, second and third trimesters, respectively. Menopause: during the menopause transition, total oestrogen levels fluctuate and gradually decline to <50 pg/mL. Progesterone levels also decline post‐menopause to <10 ng/dL. Puberty, menstrual cycle, hCG and menopause hormone values are from Labcorp (2021). E2 and progesterone pregnancy hormone values are from Schock et al. (2016). E1, oestrone; E2, oestradiol; FSH, follicle‐stimulating hormone, LH, luteinizing hormone; hCG, human chorionic gonadotropin.

Puberty in females typically begins between the ages of 8 and 13 (Pinyerd & Zipf, 2005) and can be broken down into different stages: thelarche, the development of breast tissue; pubarche, the development of pubic and axillary hair; and menarche, the onset of menstruation. These changes are primarily driven by hormones (Figure 1) that result in maturation of the reproductive systems including ovarian development, increase in uterus size and vaginal changes (Breehl & Caban, 2018). The initiation of puberty is under hypothalamic control and is due to the secretion of gonadotropin‐releasing hormone (GnRH). Neuropeptides play a role in the initiation of secretion of GnRH such as kisspeptin, which is modulated by neurokinin B and dynorphin (Spaziani et al., 2021). The increased concentrations of the GnRH result in the release of luteinizing hormone (LH) and follicle‐stimulating hormone (FSH) via gonadotropic cells of the anterior pituitary gland (Whitlock et al., 2006), which affect the theca and granulosa cells of the ovary. The ovarian follicles synthesize and secrete large amounts of oestrogens, with the most prevalent being oestradiol (E2), along with progesterone and androgens such as testosterone. Additionally, increased oestrogen levels cause the lactiferous duct system to develop, while increased progesterone levels trigger the lobular alveoli to increase in number (Pillay & Davis, 2022). Alternatively, increased testosterone levels produce axillary hair growth approximately 2 years following thelarche (Breehl & Caban, 2018). Additionally, increased FSH and LH result in the first menstrual period (menarche). The onset of menarche is accompanied by an approximate 28‐day cycle of hormone changes in FSH and LH from the anterior pituitary and oestrogen and progesterone from the ovaries, which regulate the ovarian and uterine cycles, respectively, often collectively referred to as the menstrual cycle (Figure 1). Subsequently, increases in plasma E2 concentrations cause uterine endometrium growth (Thiyagarajan et al., 2022), while elevations in progesterone prepare the endometrium for implantation by increasing the thickening of the endometrial lining (Sundström‐Poromaa et al., 2020). Importantly, these hormonal changes during puberty and throughout the ovarian and uterine cycles have effects on vascular function that mediate changes in blood pressure, autonomic function, cerebral blood flow (CBF), endothelial function and arterial stiffness.

### 2.2 Blood pressure and autonomic function during puberty

**FIGURE 2:**
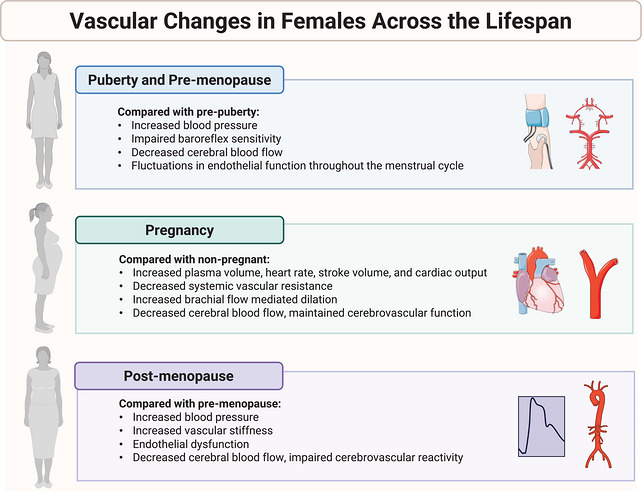
Vascular changes in females across the lifespan. Summary of vascular changes that occur during puberty and pre‐menopause (top), pregnancy (middle) and post‐menopause (bottom).

During puberty, blood pressure increases dramatically and can surpass values achieved during full maturity (Figure 2) (Li, Dong, et al., 2021; O'Neill et al., 2022; Wühl et al., 2002). As mentioned, this elevated blood pressure may be mediated by the hormonal changes that occur during puberty, specifically increases in androgens and insulin‐like growth factors, though the exact mechanisms for puberty‐related changes in blood pressure are uncertain and require additional research. Long‐term elevations in androgens in both males and females can result in vasoconstriction. For example, testosterone promotes the release of catecholamines (adrenaline and noradrenaline) that increase vascular tone (Kumai et al., 1995). Testosterone can also promote the release of endothelin‐1 from endothelial cells and increase thromboxane A2 receptor expression, both of which increase vasoconstriction (Matsuda et al., 1994; Roşca et al., 2021; Teoh et al., 2000). As such, circulating testosterone levels have been demonstrated to be positively associated with systolic blood pressure (SBP) in both boys and girls (Zhang et al., 2019). Moreover, oestrogens are the main hormones that drive puberty in females. Typically, E2 has been shown to have a blood pressure‐lowering effect (Stachenfeld, 2014) and this may contribute to why blood pressure changes that occur during puberty are lower in girls than boys. Indeed, oestrogen has been described as ‘cardioprotective’ due to both its beneficial effects on lipid metabolism and its direct interaction with the vasculature. Specifically, oestrogen modulates endothelial cells via binding to oestrogen receptors leading to increased endothelial nitric oxide synthase (eNOS) activation and nitric oxide (NO) production (Caulin‐Glaser et al., 1997; Miller & Duckles, 2008; Xing et al., 2009). This increased NO production mediates the blood pressure‐lowering effect of oestrogen. E2 can stimulate the synthesis of angiotensin in the liver, resulting in increased aldosterone that increases sodium and fluid retention (Wójcik et al., 2023). Alternatively, increased progesterone leads to sodium loss and plasma renin activity, resulting in angiotensin II and aldosterone increasing as a compensatory effect (Oelkers, 1996). As such, hormonal changes associated with puberty may play a role in the observed increases in blood pressure via increased vascular tone and total peripheral resistance until reaching adult values at the end of puberty. However, this increase in blood pressure with puberty is not pathophysiological, and pre‐menopausal females typically demonstrate lower blood pressure than age‐matched males until menopause (O'Neill et al., 2022).

These changes in hormones with puberty not only affect blood pressure via hormonal changes but also can influence autonomic function. For example, puberty can increase the incidence of syncope or presyncope in adolescent females (Walsh, 2001). Puberty is associated with orthostatic intolerance due to impaired baroreflex sensitivity as a result of increased progesterone levels, particularly in adolescents with vasovagal syncope or postural orthostatic tachycardia syndrome (Coupal et al., 2019). A metabolite of progesterone, 3α‐hydroxy‐dihydroprogesterone, has been shown to attenuate baroreflex sensitivity (Masilamani & Heesch, 1997).

### 2.3 Blood pressure and vascular function over the menstrual cycle

Changes in blood pressure during the menstrual cycle are controversial. Some studies report fluctuations in blood pressure over the menstrual cycle, such that SBP and diastolic blood pressure (DBP) are highest during menstruation, DBP is higher in the follicular phase compared to the luteal phase, and blood pressure is the lowest during days 17–26 compared to the rest of the cycle (Dunne et al., 1991). However, other studies have reported that fluctuations in blood pressure may be negligible (<2 mmHg) between menstrual phases (Drižienė et al., 2008; Thakrar, 2024).

There is clear variation in endothelial function over the menstrual cycle. For example, flow‐mediated dilatation (FMD), a measurement of endothelial‐dependent vasodilatation, has been demonstrated in young women to be elevated during the late follicular phase compared to the early and late luteal phases due to higher E2 levels that increase eNOS and subsequently enhance NO production (Brandão et al., 2014; Williams et al., 2020, 2001), while elevated levels of progesterone during the early luteal phase contribute to decreased endothelial function (Brandão et al., 2014). Moreover, whole body arterial compliance has been shown to be elevated during the late follicular phase (Williams et al., 2001). However, during the luteal phase of the menstrual cycle, elevations in both progesterone and oestrogen result in no change in baroreflex sensitivity, as oestrogen enhances baroreflex sensitivity (Brunt et al., 2013). In addition, hormonal changes over the menstrual cycle may also impact physical activity‐induced effects on vascular function (Green et al., 2016).

### 2.4 CBF changes during puberty and the menstrual cycle

In children, CBF increases with age (approximately 3.2 mL/100 g/min per year) and peaks after the age of 7 (>60 mL/100 g/min) (Paniukov et al., 2020). Subsequently, CBF begins to decrease with puberty and into adulthood (Wu et al., 2016). However, it has been demonstrated that although the decline in CBF is similar in males and females during early puberty, during the mid‐pubertal period, CBF continued to decline in males but increased with age in females (Satterthwaite et al., 2014). The authors speculated that this sex‐specific increase in CBF observed in females during puberty was a result of the increasing oestrogen levels (Satterthwaite et al., 2014). For example, it has been demonstrated that elevated oestrogen levels result in increased CBF, as oestrogen vasodilates cerebral vessels (Belfort et al., 1995; Shamma et al., 1992). Nonetheless, following puberty, CBF begins to decrease into adulthood (Figure 2).

Cerebrovascular function is also influenced by the menstrual cycle in premenopausal females and is discussed in detail elsewhere (Peltonen et al., 2016). Briefly, increased levels of oestrogen lower cerebrovascular impedance (Krejza et al., 2004) and resistance (Krejza et al., 2003), while increasing CBF (Krejza et al., 2001). As such, the highest CBF is observed during the late follicular phase of the menstrual cycle (Krejza et al., 2001). Additionally, increased oestrogen levels are associated with increased CBF during the late follicular phase of the menstrual cycle as demonstrated in women undergoing in vitro fertilization treatment (Nevo et al., 2007). Lastly, the increased CBF during the late follicular phase when oestrogen levels in premenopausal women are highest may be mediated by cyclooxygenase, which is an important control mechanism for CBF (Peltonen et al., 2016).

### 2.5 Influence of hormonal contraceptives on vascular function

Vascular function measurements are typically conducted during the placebo phase of oral contraceptives due to the known influence of oestrogen and progesterone on endothelial function in premenopausal women. For example, second‐generation oral contraceptive pills (levonorgestrel) have been shown to decrease FMD compared to naturally cycling controls (e.g., 6.4 ± 2.2% vs. 8.7 ± 3.4%) (Heidarzadeh et al., 2014; Lizarelli et al., 2009), and decrease FMD from the placebo phase to the active phase (Torgrimson et al., 2007). Additionally, progesterone‐only birth control injections (depot medroxyprogesterone acetate) have also been demonstrated to decrease FMD compared to naturally cycling controls (e.g., 1.1 ± 3.0% vs. 8.0 ± 4.8%) (Lizarelli et al., 2009; Sorensen et al., 2002). However, there are some nuances due to progesterone type, ratio of ethinyl E2 to progestin, dose and route of administration that affect the influence of hormonal contraceptives on vascular function, which are discussed in detail elsewhere (Williams & MacDonald, 2021).

Generally, oral contraceptives do not cause structural changes to the vasculature, although vascular function can be impacted as discussed above. There is limited evidence that oral contraceptives may increase the carotid artery intima–media thickness by 10% (Franceschini et al., 2013). Most studies demonstrated no effects of oral contraceptives on arterial stiffness (Enea et al., 2021; Priest et al., 2018; Yu et al., 2014). However, one study demonstrated a small increase in arterial stiffness with oral contraceptive users compared to naturally cycling controls (Hickson et al., 2011).

## 3 IMPACT OF EXERCISE ON VASCULAR FUNCTION DURING PUBERTY AND OVER THE MENSTRUAL CYCLE

**FIGURE 3:**
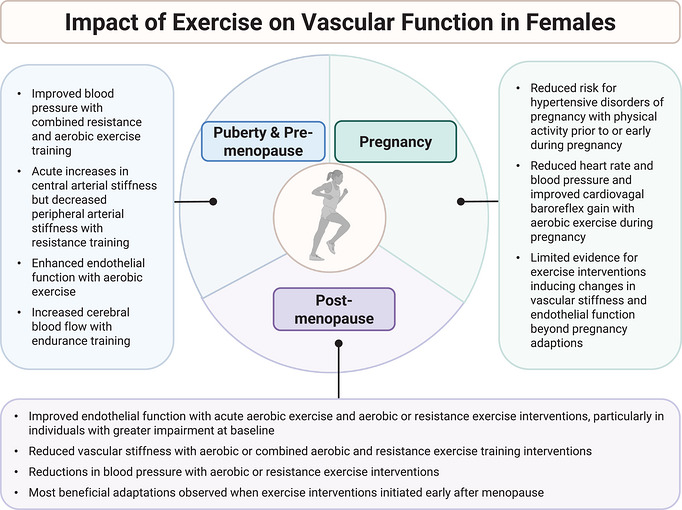
Impact of exercise on vascular function in females. Summary of the impact of exercise on vascular function during puberty and pre‐menopause (left), pregnancy (right) and post‐menopause (bottom).

Children and adolescents aged 6–17 years should complete 60 min of physical activity daily of moderate‐to‐vigorous intensity aerobic exercise. Additionally, these recommendations include muscle‐strengthening exercises at least 3 days per week and bone‐strengthening exercises at least 3 days per week (Piercy et al., 2018). The benefits of exercise on cardiovascular function in adolescents are described in Figure 3.

Interestingly, in 13‐year‐old girls increased physical activity was not associated with endothelial function but was in 13‐year‐old boys (Pahkala et al., 2008). However, when physical activity levels were matched between the girls and boys, a significant positive association with endothelial function became apparent, indicating that the lack of association between physical activity and endothelial function was due to lower physical activity levels rather than sex differences (Pahkala et al., 2008). Another study demonstrated that increased physical activity (via cycling) in girls was not associated with reduced levels of arterial stiffness; however, increased cycling was associated with lower levels of arterial stiffness in boys (Ried‐Larsen et al., 2015). Importantly, the authors did not control for the phase of menstrual cycle, which may have increased the variability in the carotid compliance measurement and confounded observations (Ried‐Larsen et al., 2015). In prepubertal children aged 6–8 years, poor cardiorespiratory fitness was independently associated with elevated arterial stiffness and impaired endothelial function (Veijalainen et al., 2016). Additionally, it has been demonstrated that endurance trained male adolescents have higher CBF compared to untrained counterparts, but this training effect was not present in female adolescents (Talbot et al., 2023). This study also demonstrated that trained prepubertal children (both males and females) have increased cerebrovascular reactivity to carbon dioxide compared to untrained counterparts (Talbot et al., 2023). These findings suggest that female sex hormones (e.g., oestrogen) impact the relationship between physical activity and both peripheral vascular function and cerebrovascular function.

Training loads with premenopausal female athletes can impact vascular function. For example, college‐aged female track athletes have demonstrated impaired endothelial function (via FMD) accompanied by decreased levels of testosterone and elevated cortisol concentrations compared to age‐matched controls (Grandys et al., 2023). Similarly, collegiate female swimmers demonstrated reduced endothelial function after 4 weeks of high‐volume training with improvements observed following a 3‐week taper (Weihl & Van Guilder, 2021). Moreover, the increased ratio of testosterone to cortisol was positively associated with increased FMD (Grandys et al., 2023). As such, overtraining that may occur in female athletes can impair endothelial function. These findings are in agreement with studies demonstrating decreased endothelial function in amenorrhoeic athletes (Augustine et al., 2016; Zeni Hoch et al., 2003). One study suggested the decreased endothelial function in amenorrhoeic athletes was associated with an unfavourable lipid profile (Rickenlund et al., 2005). Additionally, acute resistance exercise has been shown to increase central arterial stiffness and lower peripheral arterial stiffness during both the early follicular and early luteal phases of the menstrual cycle (Augustine et al., 2018). Importantly, additional research is necessary to determine the effects of overtraining on vascular function and its influence on hormones in premenopausal females and adolescents.

In obese adolescent girls, a combined resistance and aerobic exercise intervention for 12 weeks was effective at improving blood pressure and arterial stiffness (Son, Sung, Bharath, et al., 2017). Moreover, a 12‐week jump rope exercise programme has been shown to improve blood pressure and arterial stiffness in prehypertensive (defined as 120–140 mmHg SBP and/or 80–90 mmHg DBP) adolescent girls (Sung et al., 2019). Additionally, in adolescents with type 1 diabetes, 18 weeks of aerobic exercise training has been demonstrated to be effective at improving endothelial function (FMD) (Seeger et al., 2011). Collectively, these results demonstrate that in healthy adolescent females, exercise intensity, training load and menstrual cycle should be considered when studying indices of vascular function. Adolescent females with conditions such as obesity, prehypertension or metabolic disorders (diabetes) can clearly benefit from exercise interventions to improve vascular function.

## 4 HORMONAL CHANGES AND VASCULAR HEALTH DURING PREGNANCY

### 4.1 Hormonal changes during pregnancy

Pregnancy elicits vast changes in hormone levels (Figure 1). An average pregnancy is 40 weeks and is typically characterized into three trimesters of approximately 13 weeks each. Human chorionic gonadotrophin (hCG), oestrogens and progesterone are secreted by the corpus luteum during the first trimester. Hormone production is gradually taken over by the placenta at approximately week 12. One of the first detectable hormonal changes is hCG. During the first trimester, hCG quickly rises, doubling every 3 days. It peaks circa week 10–12, then declines during the remainder of the pregnancy (Friis Petersen et al., 2019; Li, Zhang, et al., 2021; O'Leary et al., 1991). The key role of hCG is to stimulate the production of sex hormones, support embryonic and fetal development and promote angiogenesis in uterine vasculature (Cole, 2010). Progesterone levels gradually increase throughout pregnancy, starting with a 10‐fold increase during the first trimester, doubling again during the second trimester, and almost tripling again during the third trimester for a total of an estimated 400% increase (Schock et al., 2016). Progesterone suppresses the gonadotropins FSH and LH, promotes uterine development essential for proper implantation of the embryo, and inhibits uterine contractions to prevent preterm birth, except during late gestation (approximately week 36), when progesterone levels plateau and slightly decrease to allow labour to progress (Kumar & Magon, 2012).

Pregnancy is associated with drastically increased oestrogen levels. Both oestrone (E1) and E2 start as androgens secreted by the maternal and fetal adrenal glands and are converted into oestrogens at the placenta. Oestriol (E3) is derived from the conversion of E2 and becomes the dominant oestrogen during pregnancy (Parisi et al., 2023). During the first trimester, E2 increases 25‐fold compared with non‐pregnancy levels, then quadruples again during the second trimester, and doubles again during the third trimester for a total of an almost 900% increase (O'Leary et al., 1991; Schock et al., 2016). Oestriol levels increase by a factor of 20, with levels peaking in the third trimester (Settiyanan et al., 2016; Yaron et al., 1999). Oestrogens prevent further ovulation by suppressing the gonadotropins FSH and LH, promote fetal development and viability, and stimulate the growth of the maternal tissues necessary to harbour the growing fetus (Parisi et al., 2023). Testosterone levels also increase almost two‐fold during pregnancy (O'Leary et al., 1991). In summary, pregnancy is associated with vast changes in sex hormones to support fetal and maternal development, particularly large increases in oestrogen and progesterone.

### 4.2 Blood pressure and vascular function during pregnancy

As pregnancy progresses, there are remarkable maternal cardiovascular and haemodynamic adaptations to accommodate maternal and fetal needs (Figure 2). Overall, these adaptations facilitate placental perfusion, provide a reserve against potential blood loss, and allow for greater blood flow distribution to the placenta, kidneys and skin to support fetal and maternal growth and temperature control (Fu & Levine, 2009; Sanghavi & Rutherford, 2014). The inability of the cardiovascular system to adapt during pregnancy may expose underlying pathology and result in pregnancy complications. For example, gestational hypertension is defined as evidence of *de novo* elevations in blood pressure greater than 140 mmHg SBP and/or greater than 90 mmHg DBP that occur after 20 weeks of pregnancy (Gynecologists, 2020). Over 50% of patients who develop gestational hypertension may develop signs of preeclampsia, which include *de novo* elevations in blood pressure along with significant end‐organ damage with or without proteinuria (Boeldt & Bird, 2017). Hypertensive disorders of pregnancy are associated with fetal and maternal vascular dysfunction that may result in preterm birth. Although the disorder typically resolves postpartum, individuals with a history of preeclampsia have a lifetime increased risk for CVD including hypertension (Bokslag et al., 2016). Therefore, adequate vascular adaptations are essential for healthy fetal and maternal development during gestation and beyond.

During pregnancy, plasma volume expansion begins at approximately 6 weeks of gestation, increasing from 10% to 15% and reaching 50% above non‐pregnancy volume during the third trimester (Chapman et al., 1998; Chesley, 1972). This is also accompanied by an increased red blood cell production, though total haematocrit is still reduced causing a physiologic anaemia (Horowitz et al., 2013). Cardiac output also undergoes large, non‐linear changes. As early as 6 weeks of gestation, cardiac output begins to increase and reaches its peak in the third trimester, 30% above non‐pregnant values (approximately 1.5 L/min greater than non‐pregnant values), then decreases slightly at the end of the third trimester (Meah et al., 2016). The early increases in cardiac output are likely due to changes in stroke volume, as stroke volume increases by approximately 8% in the first trimester and continues to a 13% increase (approximately 10 mL) by term (Meah et al., 2016). Later cardiac output changes are associated with heart rate, as heart rate at rest begins to increase during the first trimester, and peaks during the middle of the third trimester to term, with an average increase of 13 bpm (Clapp & Capeless, 1997; Mahendru et al., 2014). Along with increased cardiac output, there are also associated increases in minute ventilation of approximately 50% during the first trimester to accommodate higher maternal and fetal metabolic demand (Weinberger et al., 1980).

In addition to changes in plasma volume, there are substantial decreases in systemic vascular resistance to accommodate the needs of the uteroplacental circulation, which requires high flow and low resistance. Systemic vascular resistance decreases at 5 weeks of gestation and gradually decreases throughout pregnancy, with the nadir about 30% lower than non‐pregnant levels early in the third trimester (Meah et al., 2016). Decreased systemic vascular resistance has been attributed to a reduction in the baroreceptor set point (Greenwood et al., 2001; Tkachenko et al., 2014), reduced neurovascular transduction despite elevated muscle sympathetic nervous system activity (MSNA) (Brislane et al., 2023; Fu & Levine, 2009; Greenwall et al., 2023; Reyes et al., 2018), and decreased responsiveness to angiotensin II despite increased plasma renin and aldosterone (Chapman et al., 1998; Tkachenko et al., 2014).

In an uncomplicated pregnancy, the increased plasma volume accompanied by the decreased systemic vascular resistance results in either a reduction, albeit small (less than 10%), or no change in mean arterial pressure (MAP) (Loerup et al., 2019). This occurs until late in the third trimester when MAP may return to baseline, or even slightly rise above baseline levels. Local vasodilatation and vascular remodelling contribute to the reduction in systemic vascular resistance that occurs during pregnancy. NO, a potent vasodilator, and eNOS are upregulated during pregnancy (Nelson et al., 2000). Higher NO is likely produced by increased shear stress on endothelial cells caused by the plasma volume expansion accompanied by elevated cardiac output (Maul et al., 2003; Sladek et al., 1997). Also, pregnancy‐induced increases in circulating oestrogen, and upregulation of oestrogen receptors promote oestrogen‐induced endothelial vasodilatation via NO (Kakui et al., 2004; Li, Han, et al., 2021). For example, using a non‐invasive test of endothelial function, pregnancy was associated with higher FMD in the brachial artery compared with non‐pregnancy until 32 weeks’ gestation (Iacobaeus et al., 2017; Quinton et al., 2007; Seeliger et al., 2012). Decreased FMD during pregnancy is associated with gestational diabetes (Chatzakis et al., 2023) and preeclampsia (Weissgerber et al., 2016).

The central and peripheral vasculature undergoes structural remodelling to accommodate the changes in blood volume and optimize cardiac function during pregnancy. In large central arteries, the arterial diameter increases, and arterial stiffness decreases, as noted by a reduction in the β‐stiffness index in the carotid artery and a reduction in the augmentation index of the aorta (Edouard et al., 1998; Macedo et al., 2008; Mahendru et al., 2014; Osman et al., 2017; von Wowern et al., 2019). Carotid‐femoral pulse wave velocity (cfPWV), a standard measurement of central arterial stiffness, may decline throughout pregnancy, though not all studies are in agreement when a nadir is reached and some report no significant changes (Macedo et al., 2008; Phan et al., 2021; von Wowern et al., 2019). Elevated cfPWV may provide diagnostic value for hypertensive disorders of pregnancy, though more research is needed to understand if changes in arterial stiffness are a cause or consequence of gestational hypertension (Boeldt & Bird, 2017; Katsipi et al., 2014; Phan et al., 2021).

### 4.3 CBF during pregnancy

In regard to the cerebral circulation, most studies suggest that cerebral blood velocity at rest declines during pregnancy in both anterior (Belfort et al., 2001; Brislane, Jones, et al., 2020; Erkoç Ataoğlu et al., 2024; Matenchuk et al., 2020; Serra‐Serra et al., 1997; Zeeman et al., 2003) and posterior (Matenchuk et al., 2020) cerebral blood vessels. However, cerebrovascular reactivity to hypercapnia appears similar between pregnant and non‐pregnant females (Matenchuk et al., 2020; Sariri et al., 2013; Sherman et al., 2002). Furthermore, cerebral autoregulatory capacity is shifted, but maintained during pregnancy (Brislane, Jones, et al., 2020; Janzarik et al., 2014; Skow et al., 2022). Taken together, despite lower CBF at rest, cerebrovascular function remains preserved during pregnancy.

To summarize, during pregnancy, plasma volume expands, heart rate slightly increases, stroke volume increases and cardiac output increases (nonlinearly) (Figure 2). These changes are accompanied by a drop in systemic vascular resistance, and a small decrease in MAP, despite greater MSNA and activation of the renin–angiotensin–aldosterone system. CBF decreases, though cerebrovascular function is maintained. These profound circulatory and cardiovascular changes, which typically resolve after childbirth, are necessary for the developing uteroplacental circulation and the changes in maternal and fetal needs as pregnancy progresses.

## 5 IMPACT OF EXERCISE ON BLOOD PRESSURE AND VASCULAR FUNCTION DURING PREGNANCY

Major health organizations including the World Health Organization (WHO), the American College of Sports Medicine (ACSM), and the American College of Obstetricians and Gynecologists (ACOG) recommend that all pregnant and postpartum individuals without contraindications should be physically active during pregnancy, incorporating both aerobic and muscle strengthening activities throughout the week. Importantly, exercise during a healthy pregnancy is beneficial and poses minimal risks (Evenson et al., 2014). Pregnancy has been described as a ‘nine‐month stress test’ (Brislane et al., 2021) because many of the cardiovascular adaptations that occur during pregnancy are similar to cardiovascular changes observed during exercise in non‐pregnant females. It has been hypothesized that hypertensive disorders of pregnancy such as preeclampsia are caused by the inability of the cardiovascular system to adapt to the pregnant condition. In addition, exercise during the postpartum period favourably reduces maternal blood pressure (Pongpanit et al., 2024). Therefore, habitual exercise before, during pregnancy, or postpartum could lead to better maternal and fetal outcomes (as described in Figure 3) (Witvrouwen et al., 2020). This includes reductions in cardiometabolic diseases of pregnancy such as gestational diabetes and hypertensive disorders of pregnancy.

### 5.1 Impact of exercise on hypertensive disorders of pregnancy

Data from a dose–response meta‐analysis suggests that those with the highest pre‐pregnancy physical activity levels had a 20–35% relative risk reduction for pre‐eclampsia (Aune et al., 2014). In addition, data from a large observational study suggest that those who met recommended physical activity guidelines had a 50% lower risk of gestational hypertension compared with those who did not meet guidelines. However, physical activity was not related to the risk of preeclampsia (Arvizu et al., 2022). Taken together, these studies suggest that physical activity before pregnancy may be beneficial in reducing the risk for hypertensive disorders of pregnancy. Multiple meta‐analyses have addressed whether physical activity and exercise during gestation can reduce the risk for hypertensive disorders during pregnancy (Barakat et al., 2023; Davenport et al., 2018; Di Mascio et al., 2016; Giles et al., 2023; Magro‐Malosso et al., 2017; Meher & Duley, 2006). In general, these analyses report that prenatal exercise is beneficial for reducing the risk of hypertensive disorders of pregnancy and may lower blood pressure levels in individuals at risk for developing gestational hypertension. However, the magnitude of reduction in risk for hypertensive disorders of pregnancy widely varies. Importantly, none of the studies reported any safety concerns, though the development of a hypertensive disorder during pregnancy is a contraindication to exercising while pregnant. Discrepancies in findings may be related to the study population; the exercise intervention type, duration and intensity; and when during the pregnancy the intervention is performed. For example, exercise interventions early in pregnancy (first and early second trimester) may be more beneficial than later in pregnancy in reducing the risk for hypertensive disorders of pregnancy (Barakat et al., 2023; de Oliveria Melo et al., 2012; Price et al., 2012; Ruiz et al., 2013; Stafne et al., 2012). In summary, exercise before pregnancy or during early pregnancy can have beneficial effects on the risk for hypertensive disorders of pregnancy, though more research is needed to describe the dose, time and intensity of exercise required to elicit meaningful reductions in risk.

### 5.2 Impact of exercise on vascular function during pregnancy

Less is understood if the vascular adaptations to exercise are the same during pregnancy compared with non‐pregnancy. A recent meta‐analysis of randomized controlled trials comparing individuals who exercised during pregnancy with those who did not reported that exercise was associated with small reductions in heart rate (approximately 2 bpm) and blood pressure (approximately 2 mmHg) (Cai et al., 2020). During a normotensive pregnancy, moderate‐to‐vigorous physical activity was positively associated with cardiovagal baroreflex gain, suggesting that physical activity may be beneficial for reflex control of blood pressure during pregnancy (Sobierajski et al., 2018). In addition, an exercise intervention during pregnancy attenuated the rise in MSNA but did not impact reactivity to a cold pressor test (Skow, Fraser, et al., 2021). Meeting weekly moderate‐to‐vigorous physical activity guidelines during pregnancy has been associated with reduced arterial stiffness (Matenchuk et al., 2022). However, a 14‐week moderate‐intensity aerobic exercise intervention initiated at 20 weeks of a healthy, normotensive pregnancy did not elicit any significant changes in blood vessel health including carotid artery stiffness and cfPWV (Skow, Steinback, et al., 2021).

Regarding peripheral vascular adaptations, during the third trimester, moderate‐to‐vigorous physical activity was positively associated with FMD in the brachial artery (Reyes et al., 2020). In addition, a 12‐week aerobic exercise intervention improved FMD in non‐pregnant females with a history of preeclampsia (Scholten et al., 2014). However, whether an aerobic exercise intervention during pregnancy can improve FMD is controversial. One study reports improvements in FMD during pregnancy following an exercise intervention (Ramírez‐Vélez et al., 2011) though two others report no change (Boparai et al., 2021; Brislane et al., 2020a). Taken together, these studies suggest that exercise during pregnancy may be beneficial for cardiovascular regulation, though exercise interventions may not elicit measurable changes in vascular stiffness and endothelial function above and beyond changes already occurring during gestation. Further study is necessary to determine the optimal time points, durations, modes and intensities at which to deliver exercise interventions that show the greatest reduction in risk for gestational disorders. In addition, elucidating the mechanisms by which prenatal or antenatal exercise is protective against the development of hypertensive disorders of pregnancy is important, as the largest benefit may be in individuals at risk for gestational vascular disorders.

## 6 HORMONAL CHANGES AND VASCULAR HEALTH DURING MENOPAUSE

### 6.1 Hormonal changes during menopause

Middle age represents a period of significant hormonal change for females as they undergo the menopause transition (Figure 1). Natural menopause is characterized by the cessation of menstruation for a period of 12 months resulting from the loss of ovarian follicular function (Ambikairajah et al., 2022). While there is significant variability in the age at menopause, natural menopause typically occurs between 45 and 55 years of age (Lay et al., 2020) with the average age at menopause being 51–52 years of age (Te Velde & Pearson, 2002). Perimenopause, a period of neurohormonal dysregulation leading to progressive menstrual cycle irregularities, precedes menopause, and is characterized by significant inter‐individual variability in hormone patterns and marked fluctuations in FSH, oestrogen and inhibin (Burger et al., 2002; Greendale et al., 1999). Reductions in inhibin B occur early in perimenopause which contributes to elevations in FSH (Al‐Azzawi & Palacios, 2009; Burger et al., 2007) and early increases in LH have also been reported (Gracia et al., 2005). These hormonal changes are followed by a later decline in oestrogen (Gracia et al., 2005; Santoro et al., 2021). Further, during perimenopause, there is an attenuation of the rise in progesterone during the luteal phase of the menstrual cycle due to declines in follicular function and increasing anovulatory cycles (O'Connor et al., 2009). Indeed, post‐menopause, progesterone levels are non‐detectable as progesterone fails to increase (Burger et al., 2002). Post‐menopause is also characterized by oestrogen levels that are markedly reduced from premenopausal concentrations (Al‐Azzawi & Palacios, 2009). Menopause can also occur through surgical procedures at any age where the removal of the uterus or removal of both ovaries causes cessation of menstruation (Mckinlay, 1996). Removal of both ovaries also elicits abrupt declines in endogenous sex hormones, differing from the gradual declines observed during natural menopause (Al‐Azzawi & Palacios, 2009; Hendrix, 2005).

### 6.2 Blood pressure and vascular function during the menopause transition

Natural and surgical menopause is associated with an increased risk for CVD (El Khoudary et al., 2020; Rosano et al., 2007). Indeed, females who experience premature or early onset menopause are at an increased risk for CVD compared with females who undergo menopause between 45 and 60 years of age (Honigberg et al., 2019; Muka et al., 2016). This is likely mediated by detrimental changes to vascular health that occur during the menopause transition (Figure 2).

Endothelial dysfunction is one of the most marked alterations that accompanies menopause. Postmenopausal females have demonstrated lower brachial artery FMD compared with premenopausal females (Brislane, Low, et al., 2020; Moreau et al., 2020). Cross‐sectional studies present challenges in separating the impact of age and menopause on endothelial function. However, in similarly aged females across the menopause transition, reduced brachial artery FMD remained apparent in perimenopausal and postmenopausal females relative to premenopausal or early perimenopausal females (Bechlioulis et al., 2010; Moreau et al., 2012) and menopausal stage was more strongly associated with FMD than age (Moreau et al., 2012). Further, acute ovarian hormone suppression, following a GnRH antagonist, attenuated brachial artery FMD in premenopausal and perimenopausal females, which was reversed following E2 administration (Moreau et al., 2020). Taken together, these findings provide evidence for menopause‐mediated endothelial dysfunction independent of advancing age. Additionally, these studies suggest rapid changes in endothelial function that are observed early in natural menopause (Bechlioulis et al., 2010; Moreau et al., 2020, 2012). Further, brachial artery FMD was reduced 1 week following surgical menopause while no differences were observed in the sham condition (Ohmichi et al., 2003). Therefore, marked declines in endothelial function occur early during the menopause transition. In addition to endothelial dysfunction, menopause appears to accelerate the age‐related increase in arterial stiffness. Progressive declines in carotid artery compliance were reported in females across the menopausal transition (Hildreth et al., 2014), suggesting rapid changes in arterial stiffness with menopause. This finding is supported by increases in cfPWV or brachial‐ankle pulse wave velocity (PWV) (Samargandy et al., 2020; Staessen et al., 2001; Takahashi et al., 2005; Zaydun et al., 2006) which are observed within 1 year of menopause (Samargandy et al., 2020). Additionally, lower carotid artery compliance was observed in females who underwent hysterectomy alone or hysterectomy with bilateral oophorectomy when compared with females without hysterectomy (Gavin et al., 2012). Along with increases in arterial stiffness, menopause is associated with elevated blood pressure independent of age (Amigoni et al., 2000; Izumi et al., 2007; Staessen et al., 2001, 1997). Elevated blood pressure with menopause likely relates to steeper age‐related increases in muscle sympathetic nerve activity observed in females compared with males (Keir et al., 2020). Females between 40 and 60 years of age, demonstrate strong, positive associations between muscle sympathetic nerve activity and SBP while similarly aged males show weak associations (Keir et al., 2020). Indeed, greater autonomic support of blood pressure is observed in postmenopausal females than in premenopausal females (Barnes et al., 2014; Wenner et al., 2022), which contributes to elevated blood pressure during the menopause transition.

### 6.3 CBF during the menopause transition

The cerebral vasculature also undergoes changes during the menopause transition. Under resting conditions, CBF and middle cerebral artery blood velocity were lower in postmenopausal females than in premenopausal females (Brislane, Low, et al., 2020; Guo et al., 2024). However, cerebrovascular reactivity to hypercapnia and cerebral autoregulation did not differ between pre‐ and post‐menopause (Brislane, Low, et al., 2020). In contrast, we previously demonstrated that females who experienced menopause at an earlier age had lower cerebrovascular reactivity than similarly aged females who experienced menopause at a later age (Moir et al., 2023). Further, time since menopause was inversely associated with cerebrovascular reactivity (Moir et al., 2023), suggesting that declines in cerebrovascular reactivity may occur gradually following prolonged oestrogen deficiency. Moreover, shear‐mediated dilatation of the internal carotid artery (ICA) was attenuated in perimenopausal and postmenopausal females compared with premenopausal females, and positive associations were reported between serum E2 and ICA shear‐mediated dilatation that remained after accounting for age (Iwamoto et al., 2021). Therefore, menopause may impact vasodilatory responses in the extracranial arteries early whereas delayed impairments may be observed in the intracranial arteries and impaired cerebrovascular function may contribute to reductions in resting CBF.

### 6.4 Impact of hormone replacement therapy (HRT) on vascular function in postmenopausal females

HRT is medication containing female hormones (e.g., pill, patch, gel) and represents a common strategy to mitigate symptoms of menopause, including hot flashes, and improve bone health in postmenopausal females. Beyond mitigating symptoms of menopause, HRT, which is commonly oestrogen‐based, also impacts vascular function in postmenopausal females. Acute (e.g., within 18 h) and short‐term (e.g., 1–6 months) HRT interventions improved brachial artery FMD (Bush et al., 1998; Gužič‐Salobir et al., 2001; Koh et al., 2004; Mercuro et al., 2003; Sherwood et al., 2007; Vitale et al., 2008) and increased forearm vasodilatation and blood flow responses to acetylcholine (Gilligan et al., 1994; Vehkavaara et al., 2000). Importantly, HRT‐mediated improvements in endothelial function can be influenced by time since menopause (Sherwood et al., 2007; Vitale et al., 2008), type of HRT (Akman et al., 2013; Vehkavaara et al., 2000), baseline dilatory capacity (Bush et al., 1998), and the presence of CVD or risk factors (Herrington et al., 2001; Higashi et al., 2001). Nonetheless, many studies report the beneficial effects of HRT on endothelial function in postmenopausal females. In addition to improvements in endothelial function, HRT has beneficial effects on vascular stiffness. In postmenopausal females receiving HRT, common carotid artery compliance was greater, and brachial‐ankle PWV was lower than in postmenopausal females not receiving HRT (Miura et al., 2003; Moreau et al., 2003). Further, following short‐term oestrogen treatment, brachial‐ankle PWV was reduced (Miura et al., 2003) and arteriolar distensibility was improved (De Meersman et al., 1998). However, 3 years following the cessation of treatment, no differences were observed in aortic haemodynamics between placebo and HRT groups (Harvey et al., 2017).

Effects of HRT on cerebrovascular function are less studied, although beneficial effects have been reported. Postmenopausal females receiving HRT demonstrated greater cerebrovascular reactivity compared with postmenopausal females without HRT (Kastrup et al., 1998). Further, 3 years following cessation of HRT, cerebrovascular reactivity was higher in the pooled HRT group than the placebo group (Barnes et al., 2019) suggesting long‐lasting effects of HRT on cerebrovascular function. These positive changes in cerebrovascular function may mediate improvements in resting CBF as increases in ICA blood velocity (Jokela et al., 2003) and CBF (Słopień et al., 2003; Sørensen et al., 2001) have been reported, although the brain region, type of HRT, and length of treatment may influence CBF responses to HRT.

While HRT has positive effects on vascular function in postmenopausal females, some early studies demonstrated that HRT is associated with adverse CVD outcomes (Anderson et al., 2004; Rossouw et al., 2002; Sare et al., 2008). However, further investigation revealed an important timing effect whereby HRT had beneficial effects on CVD outcomes in healthy postmenopausal women who initiated treatment early following menopause (Lobo et al., 2016; Rossouw et al., 2007; Salpeter et al., 2006). In contrast, adverse effects were observed in older adults with prolonged oestrogen deficiency (Rossouw et al., 2007; Salpeter et al., 2006; Schierbeck et al., 2012). Therefore, exercise represents a promising intervention for improving or maintaining vascular health in postmenopausal females of all ages.

## 7 IMPACT OF EXERCISE ON VASCULAR FUNCTION IN POSTMENOPAUSAL FEMALES

Physical exercise has many benefits on vascular function in young adults. Yet, oestrogen may play an important role in the beneficial effects of exercise on aging females as oestrogen receptors upregulate many pathways important for vascular health (Gliemann & Hellsten, 2019). Therefore, the reduction in oestrogen following menopause may attenuate exercise‐mediated benefits in vascular function mediated, in part, by reductions in NO bioavailability, eNOS uncoupling, and elevated production of reactive oxygen species (Moreau & Hildreth, 2014; Moreau & Ozemek, 2017). Indeed, findings have shown inconsistent effects of exercise on vascular function in postmenopausal females (Brislane et al., 2022; Lew et al., 2022). However, exercise is shown to have some positive effects on vascular function in postmenopausal females (Figure 3), and it appears that the timing of initiation of exercise interventions following menopause (Gliemann & Hellsten, 2019), as well as the type, volume and intensity of exercise, may play an important role. Importantly, the menopause transition represents a critical period for females when early interventions, such as exercise, can target vascular changes that occur during this time, and aid in mitigating the development of CVD (El Khoudary et al., 2020).

Greater physical activity levels among postmenopausal females are associated with improved vascular function. Overweight postmenopausal females who were physically active demonstrated greater brachial artery FMD compared with overweight postmenopausal females who were sedentary (Sanders et al., 2015). Also, elderly postmenopausal females who were physically active demonstrated lower brachial‐ankle PWV and lower SBP when compared with similarly aged sedentary postmenopausal females (Pekas et al., 2020). In addition to lower brachial‐ankle PWV, carotid artery compliance was higher in endurance‐trained postmenopausal females compared with sedentary postmenopausal females (Moreau et al., 2006). However, during ascorbic acid infusion, carotid artery compliance increased in the sedentary females but not the endurance‐trained females (Moreau et al., 2006). These findings may suggest a beneficial effect of exercise training on oxidative stress and subsequently carotid artery compliance (Moreau et al., 2006). Further, a recent investigation reported similar brachial artery FMD, SBP, DBP and cfPWV between late premenopausal females and early postmenopausal females with similar physical activity levels (Debray et al., 2022). Taken together, these findings suggest that physical activity may protect against endothelial dysfunction and vascular stiffness that typically accompanies menopause and can improve vessel stiffness in sedentary postmenopausal females. Cross‐sectional evidence is supported by accelerometer data whereby carotid artery stiffness was inversely associated with the duration of moderate and vigorous‐intensity physical activity (Sugawara et al., 2006). Therefore, higher levels of physical activity in postmenopausal females appear to attenuate the vascular dysfunction that accompanies menopause.

Single bouts of exercise can elicit positive vascular effects in postmenopausal females. Brachial artery FMD was improved in healthy postmenopausal females 1 h following treadmill exercise at 60% of ${\dot{V}}_{O_{2}\max}$ (Harvey, Picton, et al., 2005). In contrast, brachial artery FMD was not improved in postmenopausal females 30 min following treadmill walking exercise (Serviente et al., 2016). The contrasting findings between these two studies may relate to differences in cuff placement as well as the timing of measurements post‐exercise as initial declines in FMD post‐exercise are followed by delayed improvements (Dawson et al., 2013). Further, an inverse association was observed between the increase in FMD and baseline FMD (Harvey, Morris, et al., 2005) suggesting that greater improvements with exercise are observed in those with the greatest impairment at baseline. In addition to improvements in brachial artery FMD, sedentary postmenopausal females demonstrated reductions in SBP and DBP 45 min following treadmill exercise, which was not observed in premenopausal females (Harvey, Morris, et al., 2005). Therefore, acute bouts of aerobic exercise improve vascular function in postmenopausal females.

The use of exercise training interventions in postmenopausal females has been studied extensively. In sedentary normotensive postmenopausal females, 8–12 weeks of aerobic exercise training increased femoral artery vasodilatory responses to acetylcholine and epoprostenol, while no training effects were observed in sedentary premenopausal females (Nyberg et al., 2016). High intensity interval training for 10 weeks also increased femoral artery vasodilatory responses to maximal infusion rates of acetylcholine, but this beneficial effect was only observed in postmenopausal females with hypertension and not normotensives (Gunnarsson et al., 2020). Additionally, healthy sedentary postmenopausal females assigned to moderate aerobic exercise training for 8 weeks demonstrated significant increases in FMD while no change was observed in non‐exercising controls (Akazawa et al., 2012). Low‐intensity exercise interventions as well as resistance training exercise interventions for 12–16 weeks also elicited improvements in endothelial function in postmenopausal females (Jaime et al., 2019; Merino et al., 2013; Teixeira et al., 2019) compared with non‐exercising controls (Jaime et al., 2019; Teixeira et al., 2019). However, some studies have reported no change in endothelial function with exercise training. In sedentary normotensive postmenopausal females assigned to high intensity interval training or continuous training for 2 weeks, brachial artery FMD and microvascular function remained unchanged (Klonizakis et al., 2014). However, the shorter duration of the training intervention in this study may explain the lack of endothelial function improvement. Nonetheless, 8 weeks of high‐intensity aerobic exercise training in sedentary healthy postmenopausal females greater than 10 years post‐menopause, did not alter popliteal artery FMD (Hoier et al., 2021). While this lack of improvement in popliteal endothelial function may relate to the timing of the exercise intervention post‐menopause, this finding was also extended to recent postmenopausal females who were sedentary and healthy, whereby 8 weeks of high‐intensity exercise training did not increase brachial or popliteal artery FMD (Nørregaard et al., 2023). Importantly, some studies have demonstrated an effect of baseline endothelial function in the response to exercise training which may explain the lack of an improvement in endothelial function in some studies. In sedentary postmenopausal females, 12 weeks of aerobic exercise training produced an improvement in brachial artery FMD, but this was only observed in females with the greatest endothelial dysfunction pre‐intervention (Swift et al., 2014). Indeed, following a 6‐month exercise training intervention, the change in FMD was correlated with pre‐intervention FMD (Swift et al., 2012). Therefore, baseline endothelial function appears to influence the response to exercise training. Nonetheless, many studies have demonstrated improvements in endothelial function following exercise training interventions in postmenopausal females.

Exercise training interventions also positively impact vessel stiffness and blood pressure. Postmenopausal females assigned to an aerobic or combined aerobic and resistance exercise intervention lasting 8–20 weeks, demonstrated reductions in PWV, β‐stiffness index and augmentation index or increases in large artery compliance while no change was observed in the non‐exercise control groups (Figuero et al., 2011; Ho et al., 2020; Matsubara et al., 2014; McGavock et al., 2004; Son, Sung, Cho, et al., 2017; Tanahashi et al., 2014; Wong et al., 2019). Further, aerobic exercise performed at moderate or vigorous intensities for 12 weeks produced similar reductions in the β‐stiffness index in sedentary or recreationally active postmenopausal females (Sugawara et al., 2006). However, low‐intensity aerobic or resistance training interventions for 12 weeks did not elicit reductions in PWV or augmentation index in postmenopausal females (Figuero et al., 2013; Seals et al., 2001). Therefore, moderate to vigorous intensity exercise interventions consisting of aerobic or combined aerobic and resistance training exercises reduce blood vessel stiffness. Along with these reductions in vessel stiffness, improvements in blood pressure are also observed with exercise interventions. Following 8–20 weeks of exercise training, SBP, DBP, MAP and pulse pressure were significantly reduced (Figuero et al., 2011; Figueroa et al., 2013; Jarrete et al., 2014; Mandrup et al., 2017; Seals et al., 2001; Son, Sung, Cho, et al., 2017; Wong et al., 2019) while no difference was observed in non‐exercise controls (Figuero et al., 2011; Son, Sung, Cho, et al., 2017; Wong et al., 2019). However, in sedentary healthy postmenopausal females greater than 10 years post‐menopause, 8 weeks of high‐intensity aerobic exercise training did not alter MAP (Hoier et al., 2021) suggesting an important timing effect of initiating exercise training post‐menopause. Therefore, exercise interventions can positively impact vessel stiffness and blood pressure in postmenopausal females, particularly when moderate or vigorous‐intensity aerobic or combined aerobic and resistance exercise is performed and interventions are initiated early during or following the menopause transition.

### 7.1 Influence of HRT on exercise‐mediated impacts on vascular function

Given the beneficial impacts of HRT on vascular function in postmenopausal females, HRT may augment the positive effects of exercise on vascular function in postmenopausal females. At 120 min post‐exercise, brachial artery FMD increased from pre‐exercise levels following E2 treatment while no improvement was observed following placebo treatment (Ozemek et al., 2020). Additionally, following 12 weeks of aerobic exercise training, brachial artery FMD was increased in the HRT groups while this effect was not observed in the placebo group (Moreau et al., 2013). Taken together, these studies suggest a positive impact of HRT on exercise‐mediated improvements in endothelial function. However, following acute treadmill exercise, brachial artery FMD was increased from pre‐exercise, and following 4 weeks of oral E2, brachial artery FMD was greater at rest but not further increased following acute exercise (Harvey, Picton, et al., 2005) suggesting a potential ceiling effect. In contrast, in sedentary postmenopausal females, combined aerobic exercise and HRT had favourable effects on resting DBP and resting forearm blood flow while no changes were observed with HRT alone (Binder et al., 1996; Oneda et al., 2014). Also, additive effects of exercise and HRT have been reported for blood pressure where combined exercise and HRT treatments further improved SBP compared to exercise and HRT interventions alone (Sánchez‐Delgado et al., 2023).

## 8 CONCLUSION

Throughout their lifespan, females may experience large fluctuations in sex hormones related to puberty, pregnancy or menopause that influence vascular health. During puberty, blood pressure increases to adult levels, and vascular risk in adult females remains relatively low until menopause. Menopause is associated with a decline in endothelial function accompanied by increased blood pressure and vascular stiffness. Pregnancy presents unique vascular adaptations to accommodate fetal and maternal needs, though the inability of the vasculature to adapt may result in complications such as hypertensive disorders of pregnancy. Additionally, habitual exercise is associated with reduced CVD risk in females of all ages. Increased physical activity in adolescent females improves vascular health, especially in adolescent females with obesity, prehypertension and metabolic disorders. Exercise also has beneficial effects on vascular function in healthy postmenopausal females and postmenopausal females at elevated CVD risk (e.g., sedentary, overweight, hypertensive); yet it appears that the sooner exercise is initiated post‐menopause, the greater the benefits. In addition, physical activity before or early during pregnancy can reduce the risk of hypertensive disorders of pregnancy. However, there are still many areas for further investigation. For example, studies that include both endothelial‐dependent and endothelial‐independent vasodilatation (e.g., responses to sodium nitroprusside) may provide a more comprehensive understanding of vascular health. More research is necessary that describes the impact of diverse types of exercise interventions other than aerobic training, such as resistance training, balance training or combined interventions. Future studies should assess the optimal exercise interventions to promote vascular health in females across the lifespan, including the mode, time, intensity and duration of the intervention. In addition, the potential additive effects of exercise and exogenous hormones on the vasculature in females warrant further investigation. Finally, more information is needed regarding the impact of exercise on vascular function in gender‐diverse individuals.

## AUTHOR CONTRIBUTIONS

Kathleen B. Miller, M. Erin Moir, and Brandon G. Fico conceptualized and designed the work; Kathleen B. Miller, M. Erin Moir, and Brandon G. Fico acquired, analyzed, and interpreted data for the work, and Kathleen B. Miller, M. Erin Moir, and Brandon G. Fico drafted the work and revised it critically for important intellectual content. All authors approve of the final version of the manuscript and agree to be accountable for all aspects of the work in ensuring that questions related to the accuracy of the integrity of any part of the work are appropriately investigated and resolved. All persons designated as authors qualify for authorship, and all those who qualify for authorship are listed.

## CONFLICT OF INTEREST

None declared.
